# Building core capacities at the designated points of entry according to the International Health Regulations 2005: a review of the progress and prospects in Taiwan

**DOI:** 10.3402/gha.v7.24516

**Published:** 2014-07-17

**Authors:** Hsiao-Hsuan Chiu, Jui-Wei Hsieh, Yi-Chun Wu, Jih-Haw Chou, Feng-Yee Chang

**Affiliations:** 1 Centers for Disease Control, Ministry of Health and Welfare, Taipei City, Taiwan (R.O.C.); 2 Department of Internal Medicine, Tri-Service General Hospital, National Defense Medical Center, Taipei City, Taiwan (R.O.C.)

**Keywords:** IHR 2005, core capacity requirements, points of entry, capacity building, cross-sectoral collaboration

## Abstract

**Background:**

As designated points of entry (PoEs) play a critical role in preventing the transmission of international public health risks, huge efforts have been invested in Taiwan to improve the core capacities specified in the International Health Regulations 2005 (IHR 2005). This article reviews how Taiwan strengthened the core capacities at the Taoyuan International Airport (TIA) and the Port of Kaohsiung (PoK) by applying a new, practicable model.

**Design:**

An IHR PoE program was initiated for implementing the IHR core capacities at designated PoEs. The main methods of this program were 1) identifying the designated PoEs according to the pre-determined criteria, 2) identifying the competent authority for each health measure, 3) building a close collaborative relationship between stakeholders from the central and PoE level, 4) designing three stages of systematic assessment using the assessment tool published by the World Health Organization (WHO), and 5) undertaking action plans targeting the gaps identified by the assessments.

**Results:**

Results of the self-assessment, preliminary external assessment, and follow-up external assessment revealed a continuous progressive trend at the TIA (86, 91, and 100%, respectively), and at the PoK (77, 97, and 99.9%, respectively). The results of the follow-up external assessment indicated that both these designated PoEs already conformed to the IHR requirements. These achievements were highly associated with strong collaboration, continuous empowerment, efficient resource integration, and sustained commitments.

**Conclusions:**

Considering that many countries had requested for an extension on the deadline to fulfill the IHR 2005 core capacity requirements, Taiwan's experiences can be a source of learning for countries striving to fully implement these requirements. Further, in order to broaden the scope of public health protection into promoting global security, Taiwan will keep its commitments on multisectoral cooperation, human resource capacity building, and maintaining routine and emergency capacities.

Taiwan, an island situated in the subtropical zone, economically relies on international trade and has long been aware about preventing the import of communicable diseases through the thriving international traffic. The point of entry (PoE) plays a critical role for early detection of disease risks and the mitigation of the impact of the rapid spread of infections. Therefore, border health measures have been applied at all international airports and seaports by the Taiwan Centers for Disease Control (TCDC). However, the emergence of new import infections urged Taiwan to ensure the capability of responding to the pandemic at PoEs. As it has always been a challenge to ensure prompt mobilization of various stakeholders at PoEs for jointly battling a threat of an epidemic, TCDC invested substantial efforts to seek a systematic and focused approach that can develop PoE capacities with the strong collaboration of stakeholders.

Meanwhile, as the severe acute respiratory syndrome (SARS) epidemic had challenged the traditional rationale of global health protection (1–3), the International Health Regulations (IHR) were revised and formally approved by the 58th World Health Assembly (WHA) in 2005 (therefore called International Health Regulations 2005; IHR 2005) (4). The new regulations require state parties to enhance and monitor eight national core capacities. In addition, it also extends its scope by including the responsibility to respond to zoonotic, food safety, chemical, and radiological hazards (5). The intent is to assist countries in focusing their efforts on the improvement of the detection, control, and response to international public health emergencies at their sources. Taiwan spared no efforts in conforming to the IHR 2005 provisions and announced the willingness to implement them 1 year ahead of schedule. Thus, before the IHR 2005 was officially enforced on June 15, 2007, Taiwan had coordinated relevant government authorities to adopt the IHR provisions into domestic legislations, specifically in acts such as the ‘Communicable Disease Control Act’ and ‘Regulations Governing Quarantine at Ports’. The amended legislations, which were regarded as the foundation of fulfilling IHR requirements, combined the goals and measures to be implemented in Taiwan, with the emphasis on the improvement of capacities in detecting, assessing, notifying, and responding to public health threats.

In the IHR 2005, the provisions for PoEs have been designed to minimize public health risks caused by the spread of diseases through international traffic (6). Based on the requirements stated in the Regulations, the state party need to designate specific PoEs, to develop the capacities listed in Annex 1B of the IHR 2005. In 2009, the World Health Organization (WHO) published an *Assessment Tool for Core Capacity Requirements at Designated Airports, Ports and Ground Crossings* (7). In this tool, the capacity requirements have been transformed into 95 assessing indicators, which enable state parties to identify existing capacities or potential gaps, along with the formulation of plans of action that address the capacities that need to be improved, by assessing the stage of implementation. The 95 assessing indicators can be separated into three parts: 1) Part A: communication and coordination framework among various stakeholders, 2) Part BI: capacities necessary at all times (called Routine capacities), and 3) Part BII: responding to public health events of international concern (PHEIC, also called Emergencies).

Right after the TCDC learned that the WHO assessment tool was published, the institute found that PoE requirements listed in the tool highly echoed its continuous anticipation of improving Taiwanese PoEs’ own capacities in routine preparedness and effective response to health emergencies, in a manner that avoids the unnecessary interference with international travel and trade. Therefore, the WHO assessment tool seemed very helpful in this context because it provided a concrete framework for Taiwan to identify the capacities to be examined, and the actions to be taken for achieving the goals. Subsequently, the TCDC decided to concentrate on adopting this tool in Taiwan, as soon as possible.

## Launching a new ‘IHR PoE program’

In the absence of the WHO's guidance on how to adopt this tool to develop pragmatic readiness, the TCDC collected extensive information about the expertise and experiences of other countries by website and article searches (8–11), email inquiries, and on-site visits (Canada and Australia). On the other hand, from their experience with previous pandemics, the global societies realized that a consistent policy based on common protocols and the cooperation between authorities at regional, national, and international levels are very critical for effective management of public health risks (12, 13). Similarly, as the IHR requirements for core capacities of the PoEs mainly address the public health events, many additional aspects such as financial support, human resources, PoE facilities, PoE routine activities, and so on need to be considered while making policies and developing related protocol. It also highlights the importance of close communication and collaboration between international organizations, the central and local governments, and various competent authorities at PoEs (14). It was found that meeting IHR 2005 obligations at a PoE is a universal challenge involving human resources, multisectoral engagement, and communication (15). Further, some countries developed a cross-departmental network/ platform, at either the central or PoE level, in order to facilitate the development of these core capacities (16, 17).

Based on the abovementioned findings, several important conclusions were made, which includes: 1) the designation of the PoEs should be based on consensus; 2) the stakeholders with the responsibility of implementing the core capacities should be brought together, not only from the health sector, but also from other public and private sectors; 3) the successful implementation of this program requires strong support from the cabinet and its subordinate organizations; 4) a coordination mechanism, with clear functions and structure, is necessary; 5) an agreed protocol, which clarifies the strategies, timeline, and multidisciplinary/multisectoral duties, is essential, and 6) all strategies should be harmonized with the currently available resources, national administrative structure, and consensus made by the participants.

In accordance with important conclusions drawn from the preparatory analysis, the TCDC submitted a protocol for a new program (called ‘IHR PoE program’) to the Executive Yuan. Subsequently, through extensive review and discussion, the protocol was formally approved to be implemented in January 2011.

### Identifying designated PoEs

After consulting various authorities, four criteria were taken into consideration while identifying designated PoEs: 1) the number of international conveyances, 2) the number of international travelers, 3) the geographical distribution, and 4) the existing facilities and logistic capabilities. Thus, one airport (the Taoyuan International Airport; TIA) and one seaport (the Port of Kaohsiung; PoK) were consensually designated at the first stage.

Every year, over 70% of the travelers enter Taiwan through the TIA. The airport has experienced a remarkable annual increase in the number of international travelers and aircraft during recent years, and an additional terminal is being planned. The PoK, which is the largest seaport in Taiwan, traditionally dealt with freight transportation. However, in the past few years, it is also being used to harbor cruise vessels and has shown a rising trend in the number of arriving travelers. In addition, a new travel center is currently being constructed. It is noted that, to upgrade competencies and promote the development of the TIA and PoK, during 2010–2012, the responsibility of operation and management of both the PoEs was shifted from the government authority to the state-run corporations. Since then, these state-run corporations have been responsible for the coordination and consolidation of various agencies stationed in the designated PoEs, under the supervision of the Ministry of Transportation and Communications (MOTC) of the central government.

### Identification of a competent authority

Since the beginning of the implementation of the IHR PoE program, there has been an interesting discussion on the competent authority at the designated PoEs. According to the IHR 2005 definition, a competent authority means ‘an authority responsible for the implementation and application of “health measures”’. Originally, it was perceived that the TCDC shall serve this role. However, when the core capacity requirements were examined in detail, it was realized that some of the health measures mentioned in IHR 2005 were being conducted by other government authorities in reality. For example, if an ill traveler with symptoms of a possible communicable disease was reported in the PoK, the TCDC will be responsible for the assessment of the traveler. If the TCDC decided to refer the traveler to a hospital for further diagnosis and treatment, the PoK fire brigade would be responsible for transporting the traveler to the hospital (with their trained personnel and equipment). Thus, in order to cater to the practical situation in Taiwan, it was consensually decided that the competent authority shall vary according to the different health measures.

### Establishment of a bidirectional coordination mechanism

#### At the PoE level

After the SARS outbreak in 2003, the importance of enhancing the cross-sectoral involvement through an integrated coordinative structure at PoE level was highlighted. Subsequently, while implementing the IHR 2005, each PoE in Taiwan established a ‘port sanitary group’ as a network of various stakeholders. The group comprised authorities from departments responsible for customs, immigration, quarantine, security and port management, and conveyance operators, as well as the local health authorities. It has successfully served as a coordinating platform at PoE level, targeted to the preparedness and the effective contingency arrangement of port health matters. During the H1N1 pandemic in 2009, the port sanitary group functioned very well on efficient information sharing, policy declaration, and coordination about any public health measures carried out at the PoEs.

Based on the positive coordination experiences, a specific taskforce (also called PoE Taskforce) was established under the port sanitary group at the TIA and PoK to facilitate the fulfillment of the core capacity requirements. In addition to members of the port sanitary group, this taskforce also involved private sectors such as sky catering, ground handling, and cargo terminal services. The PoE Taskforce coordinated with members 1) to conduct core capacity self-assessment, 2) to develop PoE action plans that address gaps identified from the assessment, 3) to monitor the progress of the action plans, and 4) to provide feedback to the central governments on difficulties that have been identified regarding core capacity development.

#### At the central government level

The competent authorities at the PoE level in Taiwan are mainly under the supervision of central governments that are in charge of national policy making, budget, and legislation. Therefore, it is believed that while developing the PoE core capacities, it would be more efficient if any important consensus and resource integration can be achieved in advance, at the central level, especially in terms of jurisdiction clarification and financial support. Furthermore, problems that universally exist among various PoEs are often related to issues in general structure, which can never be resolved by the individual PoEs alone. In this case, problem solving must highly rely on the mobilization of resources/support from the central government to the PoEs. For example, initially, both TIA and PoK felt that they lacked the expertise/capability to cope with radiological events, or plan their response to them because the central Atomic Energy Council commissioned no affiliated units at the PoEs. Consequently, the council dispatched experts directly to the TIA and PoK, to assist them in formulating radiological emergency planning, and provided necessary training for the early detection, operation of equipment, and approach to seek external support from the national radiological response team.

**Fig. 1:**
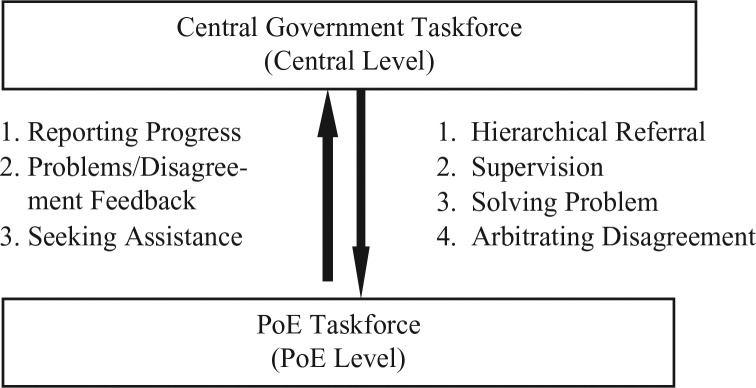
The bidirectional coordination between central and point of entry levels.

An inter-ministerial central government task force (also called Central Government Taskforce) has subsequently been constituted. Under the successive leadership of Ministers without Portfolio,^1^ 13 ministries and agencies discussed important issues such as whether to grant PoEs financial support to purchase protective equipment, when to dispatch radiological/toxic chemical experts to PoE to provide technical consultation. The taskforce supervised and regularly reviewed the progress of the designated PoEs. It can also deliver assignments and provide the PoE Taskforce policy-related and technical guidance (Fig. 1).

Additionally, authorities stationed in designated PoEs can seek immediate assistance from their superiors at the central governments, to solve problems or to arbitrate the disagreements. For instance, the TIA Co. Ltd, the government-owned company responsible for the operation and management of the airport, would like to clarify and improve current procedures related to security checks of postal parcels transported through the airport. Therefore, apart from police and custom authorities, the company also requested its superior MOTC to collaborate with the National Postal Services (also under the supervision of MOTC) in the discussion. Thus, the MOTC acted as the intermediary as well as the final decision maker (in case of any disagreements), and it was very helpful in facilitating improvement work.

## Methods

The IHR PoE program designed three systematic assessments (self-assessment, preliminary external assessment, and follow-up external assessment), together with the implementation of action plans that addressed the identified gaps. Each assessment applied the 95 assessment indicators and scoring system provided in the WHO tool (7).

### The self-assessment

Before investing resources for establishing core capacities, the PoEs must have an overall idea of its present status. Therefore, the TIA and PoK had respectively completed self-assessments during March 2011, by a PoE Taskforce. At the first stage, the secretariat of the taskforce led the introduction of the 95 assessment indicators, so that members could thoroughly understand the implication of the IHR core capacity requirements. Following this, members were asked to report their field activities concerning the IHR core capacities. In addition, a field visit was arranged for, if necessary. For instance, the TIA taskforce visited the airport cargo terminal services to understand the operation of the transportation of dangerous goods according to International Air Transport Association (IATA) regulations. Finally, members worked together to complete the WHO assessment tool, by describing any measures, facilities, and approaches available at the PoE, and listed documents which can provide evidence on the compliance of the core capacity requirements. Based on the information collected, the taskforce jointly decided the perceived stage of implementation (fully implemented, partially implemented, not implemented).

### The preliminary external assessment

Through the self-assessment, both the TIA and PoK obtained the baseline information of their existing core capacities, and then implemented some improvements to address the identified gaps. Yet, in order to avoid bias due to subjective judgments, an external expert was invited from Japan to conduct an in-depth review of the designated PoEs, from an international perspective, in August 2011. During the preliminary external assessment, the reviewer carefully verified the documents that evidenced the compliance of assessment indicators. Sites were visited to investigate facilities, equipment, and practices implemented in the field. In addition, the reviewer interviewed key personnel, raised inquiries, and obtained feedback during the assessment.

To facilitate the assessment, a pre-designed assessment protocol was provided to the reviewer and the PoEs to prepare them for the same. In addition, background materials such as self-assessment reports and a brief introduction of both the PoEs, were provided to the reviewer in advance. The reviewer was requested to pre-designate the document and the potential sites to be assessed.

### The follow-up external assessment

In order to ensure the PoE's efforts for improvement were already consistent with the IHR requirements, two experts were invited from the Australia Government's Department of Health and Ageing to undertake a follow-up external assessment in mid-March 2013. Similar assessment procedures (document reviews and on-site visits) were applied in the two external assessments to ensure that their results were comparable.

### Improvement intervention on unfulfilled core capacity requirements

**Fig. 2:**
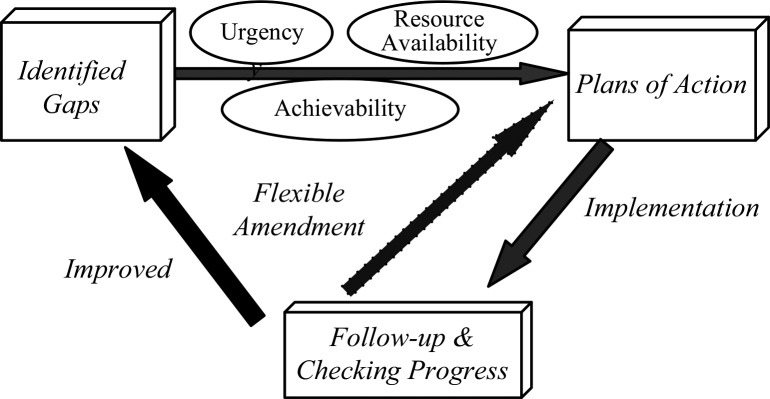
The model of strengthening core capacities at the designated points of entry.

Based on findings discovered from the self and the preliminary external assessment, a series of activities were identified to improve the designated PoEs in Taiwan. Firstly, both the PoE Taskforces respectively prioritized the identified gaps, while taking into account factors such as urgency, resource availability, and achievability. Subsequently, action plans that described practicable approaches and resource investments, and which set a defined timeline with checking points, were developed. As improvement is always a dynamic process, regular monitoring and feedback is crucial to ensure progress and that outcomes are in line with expectations. The PoE Taskforces were allowed to adjust the scope and timeline of the plans according to current need and performance (Fig. 2).

## Results

The WHO assessment tool also provided an MS Excel spreadsheet file model, which enabled reviewers to choose their response (fully implemented, partially implemented, or not implemented) to each indicator. When the performance of each indicator is determined, the model automatically generates numerical results with graphic representations of the same. According to the WHO, a PoE with a final score of above 80% is defined as fairly consistent with the requirements of the IHR Annex 1.

### Assessment results of the TIA

The TIA taskforce reported a self-assessment score of 86%. Among 89 assessment indicators (six indicators specific to seaports/ground crossing were excluded), 73.1% were assessed as ‘fully implemented’, while 26.9% were reported to be ‘partially implemented’ in the airport. It was found that the TIA owns a communication network among various stakeholders for communicable diseases or biological events, while the reporting system for radiological and chemical events had not yet been established. The airport exhibited routine capacities of adequate on-site medical services for ill travelers, and an inspection/surveillance program of the terminal environment and aircraft. However, the response plans toward radiological, chemical, and biological emergencies were not in place.

The external reviewer of the preliminary external assessment reported that 77.5% of the assessment indicators had been ‘fully implemented’, while 22.5% had been ‘partially implemented’. The total score of the TIA was 91%, which showed a fair progress as compared to the self-assessment. From the reviewer's viewpoint, the TIA had already or nearly fulfilled Part A (Communication & Coordination) and Part BI (Routine) requirements, and suggested that further efforts need to be invested in developing its capabilities to respond to PHEIC. It was noted that all the competent authorities in the TIA answered that if they encountered a problem that was difficult to address, they would consult their headquarters. However, the horizontal communication between agencies at the TIA was rarely mentioned.

Based on the learning from the previous two assessments, the TIA applied its action plans on the following areas: 1) Reconstructing the communication flow to ensure that event information is notified by and disseminated to stakeholders including outsourcing companies and service providers; 2) formulating/updating emergency plans with the consideration of the surge response. Contents of the plans, along with the communication link with various targets, were validated by table exercises or scenario drills; 3) developing regular and irregular inspection mechanisms to ensure the quality of outsourcing services, and specifying all requirements in contracts; 4) designating a specific location for the decontamination of PHEIC; 5) replacing personal protective equipments (PPEs) that had passed their expiry date, or purchasing required ones; and (6) updating standard operating procedures (SOPs) for inspecting and responding to air postal parcels.

**Fig. 3:**
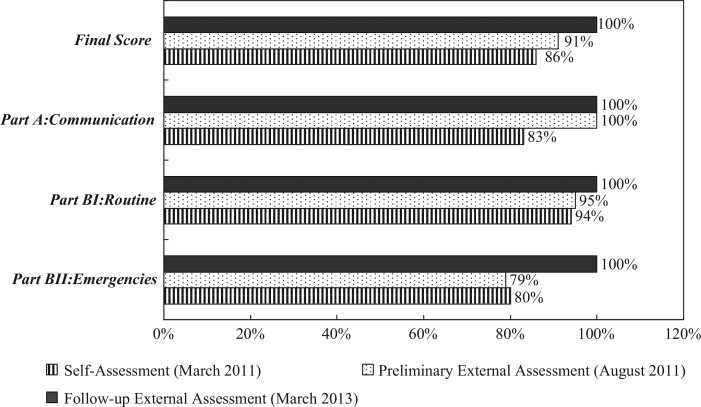
Taoyuan International Airport assessment results.

The reviewers of the follow-up external assessment reported that 100% of the indicators had been ‘fully implemented’ (Fig. 3). In general, reviewers determined five strengths of the TIA: 1) communication link with travelers for health-related information; 2) the Operation Control Center provides comprehensive infrastructure and management for PHEIC; 3) on-site medical facilities and integration of resources with contracted hospitals; 4) capacities for radiological and chemical inspection, and emergency response; and 5) providing access to, and training in the use of, PPE for all hazards. In addition, two areas of consideration were proposed in the further implementation of the core capacities. First, as the detection of fever-screening limits for respiratory diseases was an area of concern, broader surveillance parameters may be considered to enable the detection of non-respiratory diseases. Second, it was suggested that a risk assessment may be implemented to determine whether vector control measures are warranted, and whether a response protocol should be established to mitigate the risk of introduction of vectors detected in an aircraft. In response to the reviewers’ recommendations, the TCDC, as the competent authority for these matters, are carrying out policy analysis of current entry screening procedures, as well as the vector surveillance/control on aircrafts (Table 1).

**Table 1:** Key suggestions derived from three assessments undertaken in the Taoyuan International Airport

Taoyuan International Airport (TIA)	
The self-assessment (March 2011)	Establish and regularly update communication procedures during PHEIC events. Strengthen the planning of PHEIC, along with the detection, PPEs, places designated for decontamination, and training/drills necessary for responding to such emergencies. Clarify the procedure of security inspection of air postal parcels.
The preliminary external assessment (August 2011)	Ensure vertical consultation within competent authorities, as well as horizontal information sharing among competent authorities at PoE when faced by an immediate risk. A communication exercise might be necessary, especially for events with mass casualty or high profile event. As water and food services highly rely on outsourcing companies, it might be required to review documents regularly, and to conduct direct inspection by competent authorities for monitoring the sanitation. Event information should be circulated rapidly not only to public health sectors but also to the large number of service providers and outsourcing companies involved at the airport. The competent authority to be responsible for airport facilities will be expected to play a more intensive role as the control center, and it might be necessary to consider the overall appropriate ‘surge capacity’ of the TIA.
The follow-up external assessment (March 2013)	Broader surveillance parameters may be considered to enable detection of non-respiratory diseases. Undertake a risk assessment to determine whether expanded measures are warranted, and whether a response protocol should be established to mitigate the risk of introduction of vectors detected in aircrafts.

### Assessment results of the PoK

In the self-assessment, the PoK taskforce reported that 39.8% of the 88 assessment indicators (seven indicators were not applicable for seaports) had been ‘fully implemented’, 53.4% had been ‘partially implemented’, while 6.8% were evaluated as ‘not implemented’. The total score of the PoK was 77%. It was found that the communication link with senior health officials and SOPs for assessing urgent reports and disseminating information from the WHO were to be established. In addition, inspectors found a lack of knowledge about water management, swimming pool/spa, and air quality management on ships and terminal facilities. Emergency planning and PPEs toward radiological and chemical events were either absent or out of date, and a place had not been designated for decontamination.

Concerning the preliminary external assessment, the total score of the PoK was 97%. Among the 88 assessing indicators, 76.7% were assessed as ‘fully implemented’, 22.2% as ‘partially implemented’, and 1.1% as ‘not implemented’. The reviewer observed that, similar to the problems identified at the TIA, agencies in the PoK sometimes omitted the dissemination of the information to their partners in the PoEs. On the other hand, since the inspection of conveyances are sometimes closely linked with the management and the inspection of the port facilities (i.e. potable water, ballast water, and waste are transported between ships and seaport), it was appreciated that the PoK improved cross-sectional capacities by holding joint training programs for both ship and facility inspectors. In terms of emergencies, the reviewer perceived that the response system in the PoK has been improved since the self-assessment. It was also highly valued that the PoK planned ‘cross-unit human resources support programs’, which aimed to seek personnel belonging to other PoEs to support large-scale events immediately. It might help the surge capacity at the port in total.

The previous two assessments provided the PoK with clear clues to identify their action plan, which was as follows: 1) Re-examining the communication flow, taking into account private sectors such as shipping agents; 2) completing the emergency response protocols toward PHEIC. Protocols shall not only be approved by both PoE stakeholders and central authorities, but also tested by drill exercises; and 3) taking the IHR core capacity requirements into consideration while planning the new travel center, especially for terminal facilities such as water supply system, waste management, toilets, food/beverage services.

**Fig. 4:**
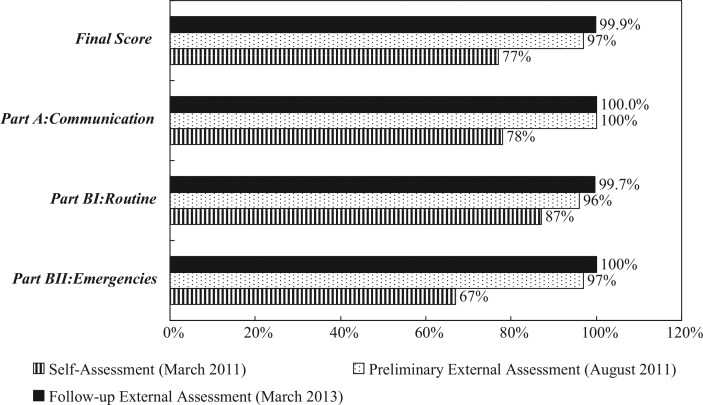
Port of Kaohsiung assessment results.

External reviewers of the follow-up assessment scored the PoK 99.9% (Fig. 4), which implied that 97.8% of the assessment indicators had been ‘fully implemented’, and 2.2% were ‘partially implemented’. It was mentioned that the SOPs and arrangements were in place with several hospitals for the diagnosis and treatment of ill travelers, and isolation, if required, including those affected by radiation, toxic chemicals, or explosives. It was noted that the PoK Disaster Mitigation and Prevention Program had been integrated to handle chemical, microbiological, and radiological emergencies. The reviewers observed the breadth of equipment and supplies used by inspection staff trained in public health risk evaluation. However, the reviewers suggested that training courses shall be developed to address risks from recreational swimming and spa areas on ships, and systems developed for the detection, assessment, and application of the recommended measures. In addition, as a new PoK travel center is underway, it was suggested that strategic workforce planning should be undertaken in anticipation of the expansion of the PoK to ensure adequate staff to implement and strengthen the IHR core capacities (Table 2).

**Table 2:** Key suggestions derived from three assessments undertaken in the Port of Kaohsiung

Port of Kaohsing (PoK)	
The self-assessment (March 2011)	Develop an inspector training program to fulfill the knowledge/skill gaps. Complete emergency response protocol, involving the decontamination planning. Replenish equipment for detection and personal protection.
The preliminary external assessment (August 2011)	In addition to notifying upper-level authorities, it is necessary to make provisions for sharing information among competent authorities at other PoEs. All authorities shall be familiarized about the agreed response protocol to handle the information according to the level of confidentiality, reliability, and indicated action determined. As construction of a new travel center is currently being planned, it will be a good opportunity to consider inclusion of capacities that make it an ideal Healthy and Safe PoE.
The follow-up external assessment (March 2013)	Development of training courses to address risks from recreational swimming and spa areas on ships, and constituting systems for detection, assessment, and application of recommended measures. As a new PoK travel center is underway, the new facility is expected to increase passenger numbers exponentially. It was suggested that a strategic workforce planning should be undertaken in anticipation of the PoK's expansion to ensure adequate staff to implement and strengthen the IHR core capacities.

Based on reviewers’ suggestions, the TCDC implemented inspection and control measures for recreational swimming and spa activities on ships into the annual training program for ship sanitation inspectors. In addition, the port management authority is increasing the number of trained inspectors, and arranging for provision of training, to increase staff knowledge.

## Discussion

In this section, we have discussed the main issues that originated from the implementation of ‘IHR PoE program’.

### TIA and PoK have met IHR 2005 core capacity requirements

**Fig. 5:**
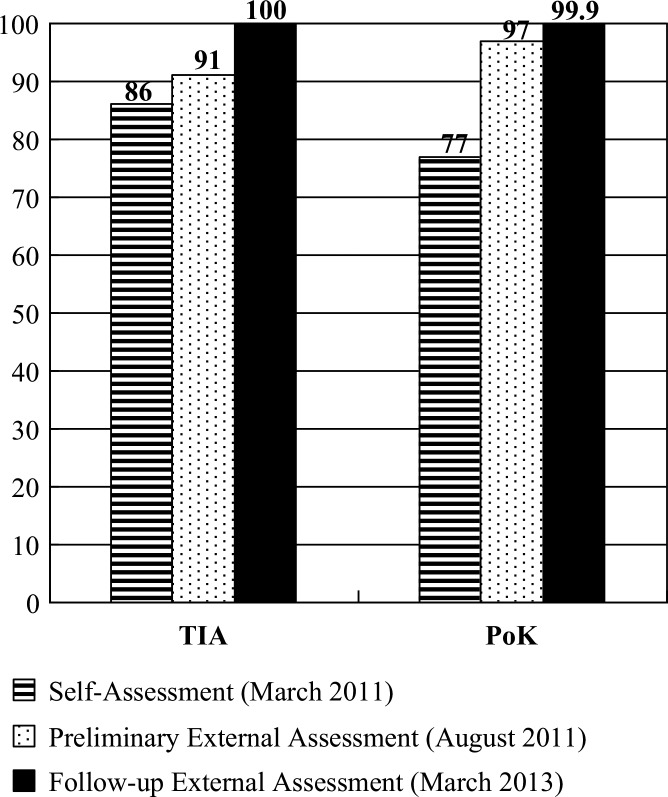
Comparison of results from various assessments of the two designated points of entry.

Results of the self-assessment, preliminary external assessment, and follow-up external assessment revealed continuously progressive trends in the TIA and PoK (Fig. 5). The follow-up external assessment (a final evaluation of this program) found that both of these designated PoEs were already highly consistent with the IHR requirements. During every assessment, it was ensured that the results were not compared between the PoEs because the TIA is a site for human travelers, while the PoK mainly handles cargo. The different situations cause different risks for human health, and need to be responded to differently. Furthermore, competition among the PoEs may lead to the perusal of higher scores, rather than the core capacity itself. Such competition should be avoided as far as possible.

As the upcoming PoK travel center, and a new TIA terminal are expected to increase passenger numbers in the next few years, existing core capacities (such as the number of skilled personnel, facilities, or surge capacity) may not be enough to handle the magnitude of travelers. It is therefore necessary to be attentive to the needs of further improvement in the core capacities through an analysis of the potential impact of the rising numbers of passengers.

### Enhancing emergency response capacity: a matter of concern

The first two assessments found that capabilities to respond to radiological and chemical events effectively were underdeveloped or nonexistent in both, the TIA and the PoK; however, the response planning for a large-scale outbreak/pandemic had been established after the SARS epidemic. These findings corroborated the evidence generated from the general review of global IHR implementation, specifically indicating that, ‘The chemical, nuclear, and radiological threats encompassed in the IHR 2005 presents a unique challenge to the surveillance and response community in both developed and developing countries’ (15).

Since the beginning of the implementation of the IHR PoE program, there was a discussion on whether radiological and chemical capacities were needed to be involved in the assessments, as the WHO ambiguously described in one of the assessment indicators (Part BI d.2.3) that ‘Harmful contamination, other than microbial contamination, such as from radionuclear sources, could also be found on ships, but is outside the scope of this guidance’. Further, due to the lack of international guidance, it seemed easier and less complicated to restrict the assessments to communicable diseases. However, it was subsequently determined that Taiwan should not ignore radiological and chemical hazards because the type of accidents that may occur at the PoEs cannot be predicted or selected. Sometimes, an accidental or deliberate release of chemical, biological, and radiological agents on arriving conveyances would have the potential to cause adverse health and financial consequences (18). In this case, imported risks cannot be identified, nor be properly dealt with, without the relevant surveillance and response capacities. The Fukushima nuclear disaster in 2011 highlighted the need to involve diversified public health risks in Taiwan. Since then, more than 200 containers imported from Japan have been detected as being contaminated in the PoK. In this case, once possible potential nuclear hazard was detected, the custom authority, authorized to undertake radiological inspections, will implement a series of SOPs including risk identification/evaluation, reporting, PPEs for staff, etc.

Through understanding the risk profile of the TIA and PoK as well as in consultation with technical experts dispatched by the central governments and national disaster response authorities, it was determined that the emergency response at PoEs level should meet the following six basic criteria: 1) PoE response protocols are aligned with those at the national and local level to ensure the seamless flow of information from the various response systems; 2) clear identification of duties and responsibilities of each stakeholder; 3) knowledge and equipment for early detection and personal protection; 4) communication mechanisms to disseminate information to relevant stakeholders at the PoEs; 5) access to external assistance from the local or central disaster response system; and 6) clear command and control system before the arrival of external assistance, and the process of command transfer.

According to the criteria defined above, the TIA and PoK updated their emergency plans, and integrated them into protocols for various types of PHEICs (19, 20). The drafts were submitted to stakeholders at the PoE and national disaster response authorities for peer-review before finalization. Meanwhile, required equipment was provided for the initial detection and personal protection (amount and type of equipment were suggested by experts from central authorities). In addition, as the success of any response is highly linked to the people who perform their roles, the required competencies and skills needed for specific response personnel were identified, and relevant training programs were designed. Regular drill exercises were conducted to verify the adequacy and interoperability of the plans, as well as to increase the stakeholders’ familiarity with their responsibilities and operational procedures. Through the abovementioned efforts, the reviewers of the follow-up assessment noted a significant progress in establishing emergency response SOPs, as well as ensuring that resources were available to ensure rapid responses. From these experiences, it was learned that despite a lack of international principles and guidance for establishing core capacities, countries should try to take its own decisions on what to do and how to do it, based on its current infrastructure, and resources and demands. In addition, where possible, countries should build on existing systems and infrastructure for strengthening their capacities, rather than immediately investing in large-scale systems or infrastructure changes.

### Active participation and strong collaboration is of the utmost importance

The advocacy of the IHR PoE program was very challenging initially. Due to a lack of understanding of the IHR 2005, key stakeholders at central ministries and PoEs perceived that the regulations were only under the jurisdiction of the health ministries. Therefore, the TCDC invested considerable effort in advocating the aim and concepts of the IHR 2005. Despite these efforts, the IHR PoE program was not implemented very smoothly, due to the resistance and complaints from the field, until the Japan Fukushima nuclear disaster occurred in 2011. Fearing the impact of nuclear contaminations (as traffic and trade are very frequent between Japan and Taiwan), people working at the PoEs were keenly aware of the importance of protecting themselves by enhancing the surveillance and response system. They found a self-motivated actor (21) to participate in developing the PoE's core capacities, and gradually realized that they were jointly contributing to something beneficial for their own workplace, safety, and health. This change of attitude was helpful for further activities. Subsequently, to protect themselves, these stakeholders were willing to think about and invest more efforts in developing capacities to address the emergency response. For instance, people were very concerned if they were to be notified about anything unusual. Therefore, they carefully reviewed and updated the contact details of every stakeholder to ensure that event information can be disseminated immediately.

It was also observed that the process of preparing for each assessment brought the stakeholders closer. As they shared the same objective of obtaining better results for the next assessment, partners involved in the PoE met frequently to discuss matters. This extensive participation of various professionals led to a robust unity and mutual understanding. The reviewers observed that all the relevant units at each PoE were united with a well-organized structure, and the contribution of the staff toward the implementation of the IHR was a major strength in the implementation of core capacities at PoEs in Taiwan. It is believed that this solidarity shaped through the IHR PoE program will continue even after the core capacity requirements have been fulfilled. It shall be continuously beneficial, not only for the daily operation of the PoEs, but also in co-confronting future crises.

Strong national efforts throughout all the governmental agencies, to achieve the core capacity requirements at the PoE, were noted by the reviewers of the preliminary and follow-up external assessments. Sometimes the application of the program was affected by bureaucracy and sectionalism, especially with reference to jurisdiction and expenditure allocation. The solution was the continuous communication and strong commitment of the higher-level authorities to carry through the program and to arbitrate contentions. Thus, Taiwan was inspired by the reviewers’ conclusions that the progress achieved is testament to the strong commitment, professionalism, and enthusiasm demonstrated by the staff, in their respective roles.

## Conclusion

This article introduced a new, but applicable, model that Taiwan developed for developing IHR core capacities at designated PoEs. The IHR PoE program focused on 1) designating two PoEs according to the pre-determined criteria, 2) identifying the competent authority for each health measure, 3) building a close collaborative relationship with stakeholders from the central and PoE levels, 4) designing and implementing three stages of systematic assessment, and 5) undertaking action plans targeting the gaps identified by the assessments. With incessant efforts invested in the ‘learning by doing’ process, the designated PoEs of TIA and PoK were assessed as highly consistent with the capacity requirements of the IHR 2005. As many countries had requested extensions to fulfill the IHR 2005 core capacity requirements (22), it is expected that experiences generated from Taiwan can provide some clues on how to identify and strengthen core capacities at designated PoEs.

Nevertheless, the achievements of the IHR PoE program do not imply an end, of a process, but are a starting point to address the increase in international traffic, and to respond to future challenges at airports and seaports effectively. As the reviewers suggested, it is necessary to continue to maintain core capacities in the designated PoEs and flexibly adjust the overall strategy in the face of new diseases (such as Middle East Respiratory Syndrome Coronavirus infection or new strains of avian flu), new terms of emergencies, new international guidance, as well as new transporting routes and destinations. For this purpose, the Taiwanese government will remain committed toward implementing and reviewing the content of the IHR PoE program. So far, the coordination and collaboration at PoEs functioned well in response to the H7N9 outbreak that began in China at the end of March 2013. The emergency of a new disease reminded Taiwan that the collaborative mechanism and the partnership, which were already successfully triggered or shaped, should be continuously maintained to ensure the sustainability of the core capacities.

Currently, Taiwanese government has launched the second stage of the IHR PoE program. Five more PoEs (three airports and two seaports) have been identified as designated PoEs to develop their core capacities. The new task will rely highly on the experiences from the development undertaken at the TIA and PoK. In addition, it will adapt to the characteristics of the specific PoEs and the ever-changing environment. These efforts are expected to extend the safety of the lives and property of Taiwanese people extensively and to bolster the competitive advantages of PoEs in Taiwan.

## Footnotes

1 In Taiwan, a Minister without Portfolio refers to a minister who does not head a particular ministry. Being the premier's key aides, such ministers are responsible in managing and reviewing the affairs of different government ministries and commissions, to enhance the competencies and governance quality of the Cabinet.
